# Case Report: Severe gastrointestinal complications in adult IgA vasculitis: a fatal case of acute esophageal necrosis

**DOI:** 10.3389/fimmu.2025.1601700

**Published:** 2025-10-21

**Authors:** Xun Zhang, Zhengbin Wu, Yuhao Li, Shifeng Shao

**Affiliations:** ^1^ Department of Critical Care Medicine, Daping Hospital, Army Medical University, State Key Laboratory of Trauma, Burns and Combined Injury, Chongqing, China; ^2^ Department of Dermatology, The First Affiliated Hospital of Chongqing Medical University, Chongqing, China

**Keywords:** Henoch-Schönlein purpura, immunoglobulin A, acute esophageal necrosis, pancreatitis, acute abdomen

## Abstract

*Henoch–Schönlein* purpura (HSP), also known as immunoglobulin A vasculitis (IgAV), is a type of systemic small-vessel inflammatory pathology. Clinical symptoms can range from simple skin purpura to multiorgan damage. IgAV can be divided into cutaneous-limited and multisystem-involved types, with significant individual variability in clinical manifestations. Between 50%–75% of patients experience abdominal symptoms such as abdominal pain and hematochezia; however, the occurrence of IgAV complicated by pancreatitis and esophageal mucosal sloughing is exceedingly rare. We report a case of adult IgAV with widespread purpura as the main clinical manifestation. Abdominal pain emerged during treatment, and pancreatitis was initially diagnosed based on serum amylase findings and CT imaging features. Nevertheless, acute esophageal necrosis was confirmed via gastroscopy, followed by a rapid deterioration in the patient’s clinical status within a short period, ultimately leading to death due to massive gastrointestinal (GI) bleeding and disseminated intravascular coagulation (DIC).

## 1 Introduction

Immunoglobulin A vasculitis (IgAV), previously referred to as hypersensitivity purpura, is the most prevalent form of systemic vasculitis in children, with an incidence rate of 70.3 per 100,000 among those aged 4–7 years, compared with an incidence rate in adults that is only one-third to one-half of that in children (1–3). IgAV is a leukocytoclastic vasculitis involving small vessels (4). Pathological examination often reveals deposits of immunoglobulin A and complement component C3 around the walls of small vessels (5). Based on varying clinical manifestations, it can be categorized into localized IgAV, which primarily affects the skin, and systemic IgAV, which involves other organs—commonly the joints, gastrointestinal (GI) tract, and kidneys (6). GI involvement in IgAV may present as gastritis, duodenitis, GI mucosal ulcers, and purpura, and can lead to severe complications such as intestinal perforation and intussusception (7).

IgAV is mainly characterized by non-thrombocytopenic purpura as the primary clinical manifestation, often accompanied by nephritis, joint pain, and abdominal pain. IgAV is an immune-mediated small-vessel vasculitis that predominantly affects children and is characterized by cutaneous purpura as the core clinical sign. Mild cases are often self-limiting, whereas some patients may experience relapses (8). Infection is a common predisposing factor for IgAV, and COVID-19 may also induce IgAV. However, the patient in this case did not present with any obvious infection-related symptoms (9). Reports of pancreatitis resulting from IgAV have been documented; however, there are no recorded instances of esophageal mucosal exfoliative manifestations associated with IgAV. This article reports a case of IgAV in an adult male in whom, during the gradual disappearance and improvement of systemic purpura, acute esophageal mucosal exfoliation and sudden massive bleeding occurred. This acute abdomen, especially with pancreatitis imaging changes, poses a challenge for clinicians in early diagnosis. By reporting this case, we aim to remind clinicians that when managing patients with IgAV complicated by abdominal pain, they should be alert to rare esophageal lesions. If necessary, early gastrointestinal endoscopy should be performed for evaluation, and early interventional treatment should be implemented to improve the patient’s prognosis.

## 2 Case report

**Figure 1:**
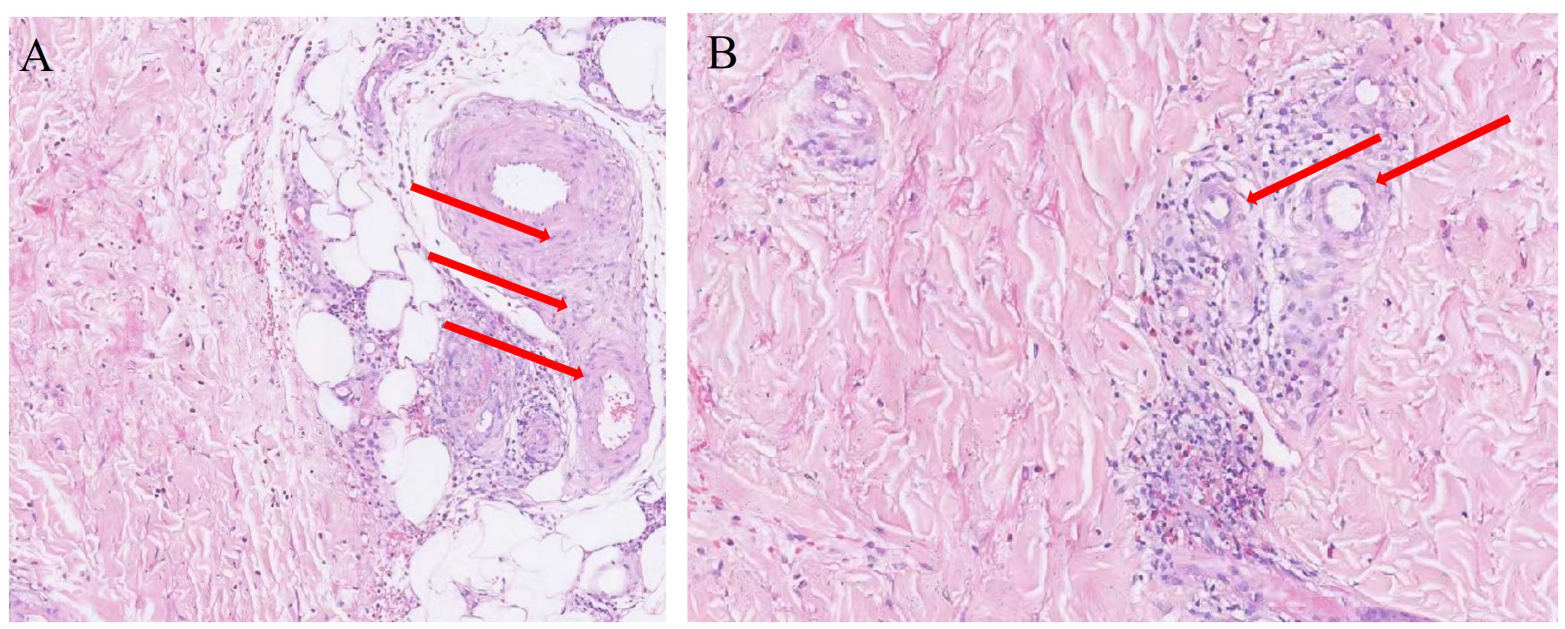
**(A, B)** Skin biopsy pathology. The areas indicated by the arrows show significant swelling of vascular endothelial cells, accompanied by extensive inflammatory cell infiltration.

A 35-year-old male developed generalized cutaneous purpura without obvious predisposing factors 20 days prior to admission, predominantly involving the face, back, abdomen, and ankle joints of both lower extremities, with a symmetrical distribution. Concurrently, he experienced diffuse epigastric abdominal pain of a cramping nature. The patient subsequently presented to the First Affiliated Hospital of Chongqing Medical University. Upon detailed inquiry, the physician identified no specific past medical, family, or medication history, and no recent signs of infection were noted. Based on the presentation of cutaneous purpura and diffuse abdominal pain, the patient was diagnosed with immunoglobulin A vasculitis (IgAV). After admission, a skin biopsy was performed, which revealed perivascular inflammatory cell infiltration and vascular endothelial cell swelling (**Figure 1**). Treatment was initiated with oral prednisone acetate tablets and cetirizine for anti-allergic therapy for 7 days. Following treatment, cutaneous purpura on the trunk resolved, abdominal pain alleviated, and only scattered purpura remained on the extremities. The patient’s condition improved, and he was discharged with continued oral anti-allergic medication after discharge.

On October 24, 2023, the patient again developed abdominal pain without obvious predisposing factors, presenting as cramping pain in the upper abdomen. The patient subsequently attended the Emergency Department of Daping Hospital, where physical examination showed stable vital signs. Mild tenderness was noted on palpation of the upper abdomen, without rebound tenderness or muscle rigidity. No abdominal distension, visible intestinal type, or peristaltic wave was observed, and no abdominal mass was palpable. The hepatic dullness boundary was normal. Scattered large patches of purpura were noted on the patient’s extremities, with fewer on the trunk (**Supplementary Figure 1**). No swelling or tenderness was found in large joints such as the knee, ankle, or elbow joints.

**Figure 2:**
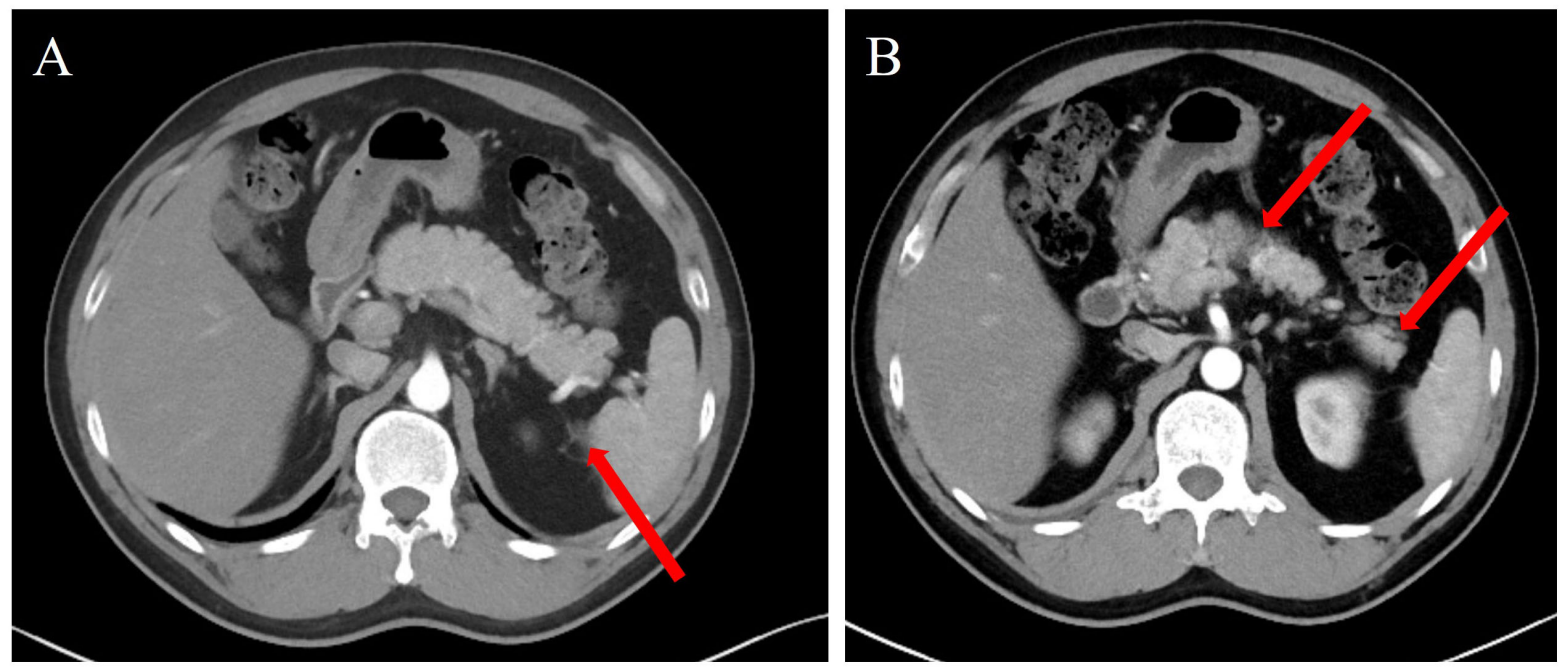
**(A, B)** Computed tomography images show pancreatic edema and surrounding inflammatory exudation. The area indicated by the red arrow demonstrates peripancreatic inflammatory exudate; however, the overall morphology of the pancreas remains intact, indicating mild pancreatitis-related injury.

Laboratory test results were as follows: white blood cell (WBC) count, 11.55 × 10^9^L; C-reactive protein (CRP), < 0.5 mg/L; platelet count, 122 × 10^9^L (mildly decreased); hemoglobin, 178 g/L; coagulation function: international normalized ratio (INR), 0.96; activated partial thromboplastin time (APTT), 28.1 s; prothrombin time (PT), 10.6 s; D-dimer, 341.68 µg/L; calcium, 0.96 mmol/L; and amylase, 124 U/L. Tumor marker tests were all negative, including those for carcinoembryonic antigen (CEA), carbohydrate antigen (CA) 19-9, and CA 50. These results indicated mild elevations of WBC and amylase (not exceeding three times the normal range). Urinary protein was not elevated, and liver and renal function showed no significant abnormalities. Abdominal CT showed mild swelling of the pancreas, with a small amount of exudation in the pancreatic tail. No segmental intestinal injury was found, and there were no signs of edematous thickening of the involved intestinal wall, free subphrenic air, intestinal stenosis, or other relevant abnormalities (**Figure 2**). The patient had definite cutaneous purpura accompanied by diffuse abdominal pain, supporting the consideration of abdominal purpura. In addition, the presence of abdominal pain combined with elevated amylase and CT findings suggestive of pancreatitis met the diagnostic criteria for pancreatitis. The patient was admitted to the Department of Gastroenterology for treatment.

The treatment regimen included fasting, inhibition of gastric acid secretion, administration of somatostatin to inhibit pancreatic enzyme secretion, antispasmodic therapy, and anti-allergic treatment with dexamethasone combined with cetirizine.

**Figure 3:**
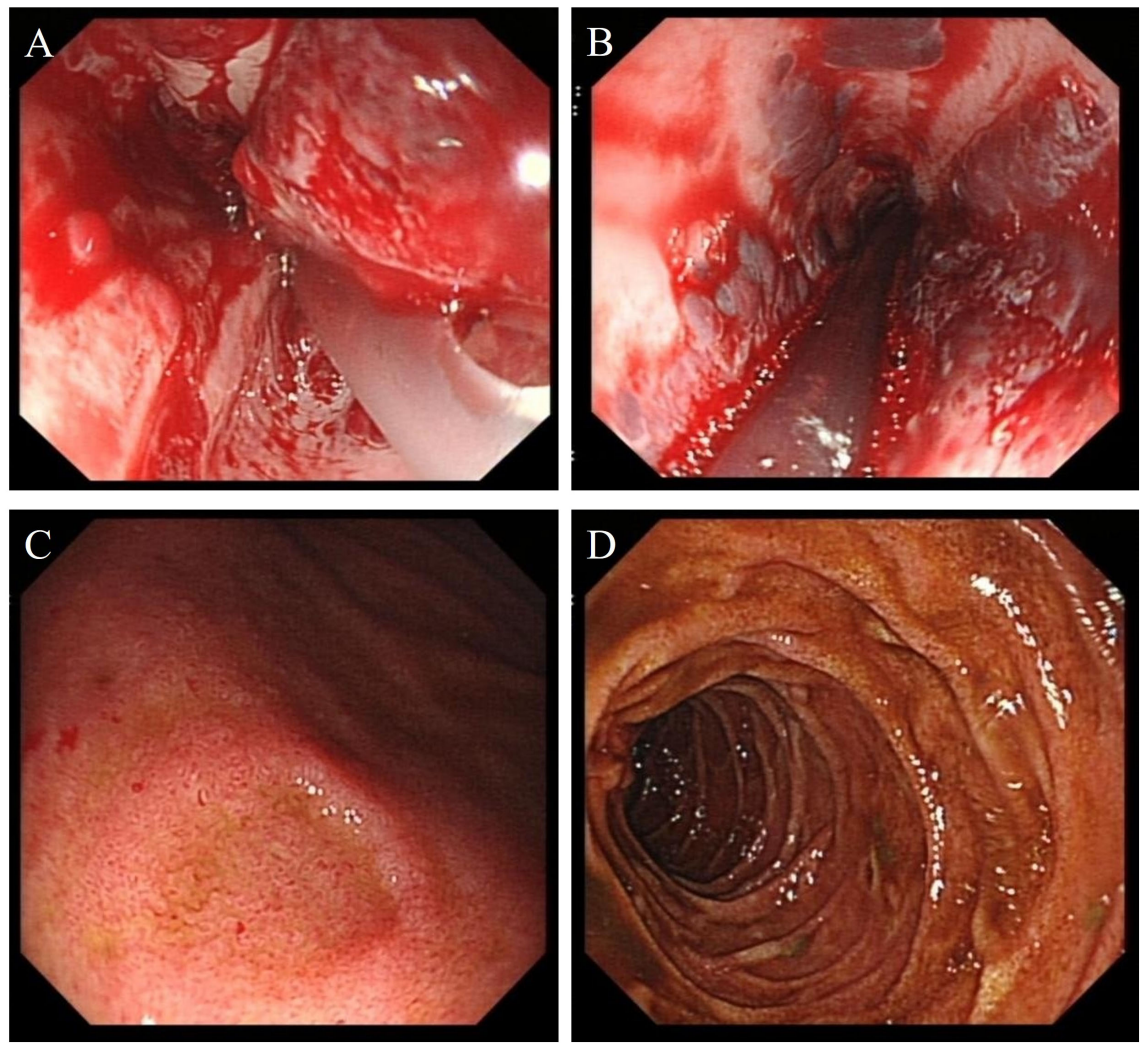
**(A, B)** Endoscopic images show features of peptic esophagitis involving the entire esophagus. **(C)** The gastric antral mucosa appears rough, with localized mucosal depression and erosion. **(D)** Scattered ulcerative lesions are visible in the descending portion of the duodenum.

**Figure 4:**
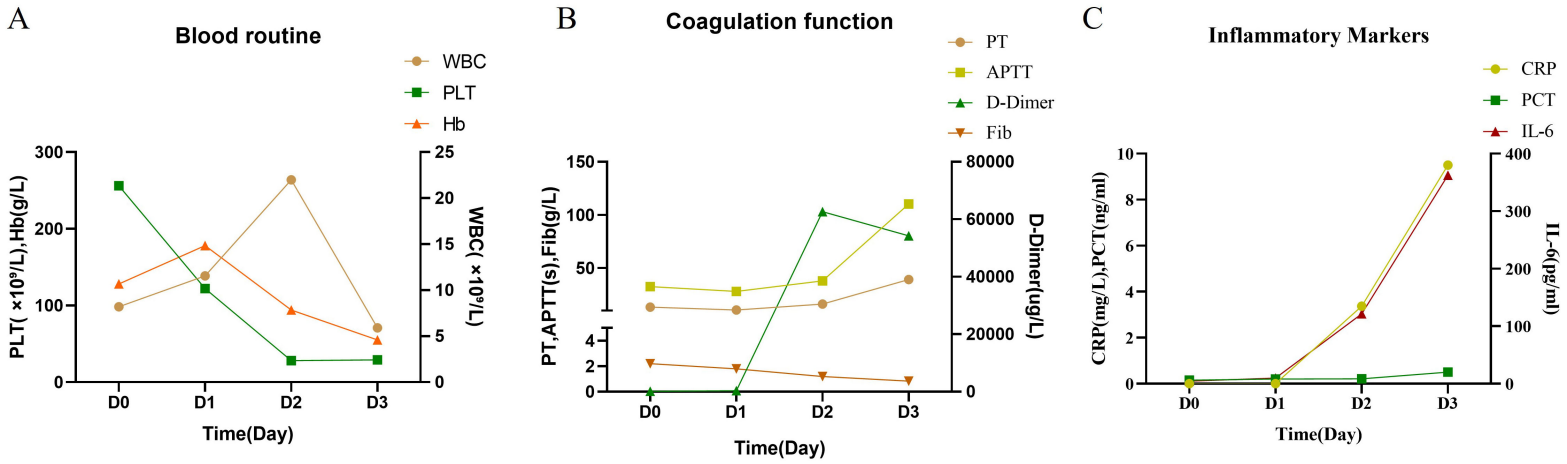
Results of laboratory tests at different time points. **(A)** Blood routine. **(B)** Coagulation function. **(C)** Inflammatory markers. WBC, white blood cell; PLT, platelet; Hb, hemoglobin; PT, prothrombin time; APTT, activated partial thromboplastin time; Fib, fibrinogen; CRP, C-reactive protein; PCT, procalcitonin; IL-6, interleukin-6.

On the second day after admission, the patient’s abdominal pain worsened, and fecal occult blood was positive. Emergency gastroscopy revealed acute peptic esophagitis involving the entire esophagus, local depression and erosion of the gastric antral mucosa, and scattered shallow ulcerative lesions in the duodenal bulb and descending portions (**Figure 3**). Laboratory tests (**Figure 4**) showed the following: WBC, 21.98 × 10^9^L; platelet count, 28 × 10^9^L; hemoglobin, 94 g/L; INR, 1.42; APTT, 38.1 s; PT [missing value—please verify]; and D-dimer, 62,624.8 µg/L. Complement C3, complement C4, immunoglobulins IgG, IgA, and IgM, autoantibody spectrum, T-lymphocyte subset analysis, procalcitonin, liver function, renal function, and urine protein tests all showed no significant abnormalities.

Bacterial and fungal smears and cultures of sputum and urine, routine stool tests, and nucleic acid screening for common viruses were all negative; thus, infections were ruled out. The test results for hepatitis antibodies, antinuclear antibodies (ANA), antineutrophil cytoplasmic antibodies (ANCA), serum complement levels, serum cryoglobulins, and rheumatoid factor (RF) were all within the normal range.

On the same day, the patient was transferred to the intensive care unit for treatment. Esomeprazole 80 mg was administered via rapid intravenous push, followed by continuous intravenous infusion of esomeprazole at 8 mg/h, along with continuous intravenous infusion of somatostatin for gastrointestinal bleeding control. Concurrently, methylprednisolone 1 mg/kg combined with intravenous immunoglobulin (IVIG) 400 mg/kg was administered intravenously.

On the third day after admission, the patient developed widespread purpura, sudden gastrointestinal bleeding, and hemorrhagic shock. Immediate treatment included blood transfusion, correction of coagulation function, conservative medical hemostasis, antimicrobial therapy, lung-protective mechanical ventilation with tracheal intubation, and fluid resuscitation. However, the patient ultimately died due to ineffective treatment of disseminated intravascular coagulation (DIC).

## 3 Discussion

The etiology of immunoglobulin A vasculitis (IgAV) remains unclear, but the formation of galactose-deficient IgA1 (Gd-IgA1) and its associated immune complexes is believed to play a significant role in the pathogenesis of systemic vasculitis and tissue damage, thus representing a crucial mechanism in the development of IgAV (10). Skin biopsy often shows leukocytoclastic vasculitis (5). Gastrointestinal (GI) manifestations are mostly ulcerative bleeding and perforation of the stomach and intestines, with severe cases such as intestinal intussusception and intestinal necrosis. The manifestation of acute esophageal mucosal exfoliation is rare (11–13). Acute stripping esophagitis is related to drugs and food, with few reported cases (14, 15). From the current literature search, no cases of IgAV concurrently complicated by acute pancreatitis and acute esophageal mucosal exfoliation have been identified.

The diagnosis of IgAV relies on clinical manifestations and laboratory findings. In this case, the patient presented with distinct cutaneous purpura and extensive peritonitis, satisfying both the 1990 American College of Rheumatology (ACR) criteria and the 2008 final Ankara classification criteria endorsed by EULAR/PRINTO/PReS. Light microscopy of the skin biopsy—a hallmark of IgAV—demonstrated prominent small-vessel vasculitis characterized by vascular wall edema, marked endothelial cell swelling, and extensive perivascular neutrophil aggregation. Admission laboratory investigations showed no thrombocytopenia and normal coagulation profiles (effectively ruling out thrombotic thrombocytopenic purpura and other hemorrhagic rash disorders), while negative antineutrophil cytoplasmic antibody (ANCA) testing excluded ANCA-associated vasculitides.

Symptoms of IgAV in adults are more severe than in children, although treatment approaches are generally similar. In this case, the patient presented with distinct abdominal pain, and CT imaging revealed pancreatic enlargement consistent with edematous pancreatitis, accompanied by elevated amylase levels, aligning with the Atlanta classification criteria for pancreatitis diagnosis (16).

In children diagnosed with IgAV, pancreatitis has been observed in a subset of cases. According to the report by Du et al., only 4 out of 15 pediatric patients exhibited amylase levels exceeding three times the normal range, with a median amylase level of 177 U/L (interquartile range 154–332 U/L; reference range 0–125 U/L). The specificity of amylase in patients with IgAV complicated by pancreatitis is relatively low, and diagnosis primarily relies on clinical manifestations and histopathological findings. Following treatment with glucocorticoids, these patients generally experienced favorable outcomes (17). Frigui summarized cases of adults with IgAV complicated by pancreatitis reported in recent years, noting that treatment with glucocorticoids yielded favorable outcomes (18). The mechanism underlying the occurrence of pancreatitis following IgAV remains unclear but may be associated with factors such as immune dysregulation or vascular occlusion (19). In our case, the onset of the patient’s symptoms occurred 7 to 10 days after the appearance of skin purpura. Amylase levels were only mildly elevated, and the pancreatic presentation was consistent with edematous pancreatitis. This suggests that the clinical manifestations of IgAV-associated pancreatitis in adults are similar to those observed in children. The cases we reported indicate that the pancreas mainly exhibits inflammatory edema. However, because the patient’s family refused an autopsy, there was no definite pathological evidence of pancreatic inflammation, and the mechanism of its occurrence could not be clearly determined.

The etiology of acute esophageal mucosal exfoliation is complex and commonly includes (1) the oral administration of certain medications, such as dabigatran etexilate and benzodiazepines; (2) ingestion of corrosive liquids; and (3) autoimmune disorders, including systemic lupus erythematosus and pemphigus vulgaris (17, 18). In adults with immunoglobulin A vasculitis (IgAV), gastrointestinal (GI) manifestations are most commonly characterized by abdominal pain, and the condition is prone to complications such as gastrointestinal bleeding. Among GI sites, the duodenum is the most frequently involved. Computed tomography typically reveals circumferential bowel wall thickening and mesenteric vascular congestion. In severe cases, patients may develop intestinal perforation or even intestinal necrosis. However, there are currently no clear cases of severe esophageal mucosal exfoliation complicated by bleeding caused by IgAV (20). Iorio et al. documented a case of a patient with allergic purpura presenting with concurrent acute esophageal necrosis who also had a history of kidney transplantation, a recognized risk factor for the development of acute esophageal mucosal exfoliation (21). Among patients with IgAV, 50%–75% experience GI involvement, with most lesions occurring in the small intestine (22–24). In our case, the patient’s condition deteriorated rapidly during hospitalization. The progression of purpura and the presence of complications such as pancreatitis indicated severe microvascular damage. In addition to the commonly observed injury to the small intestinal mucosa, extensive desquamation of the esophageal mucosa was noted. We hypothesize that the desquamation of the esophageal mucosa may be associated with ischemic necrosis of the mucosal microvasculature due to severe microvascular pathology. Although we cannot definitively establish that IgAV was the direct cause of the esophageal mucosal desquamation, the clinical presentation of the patient—together with the absence of any known risk factors for desquamative esophagitis—strongly suggests a significant correlation between the esophageal desquamation and IgAV in this case.

Glucocorticoids play a crucial role in the management of immunoglobulin A vasculitis (IgAV), particularly in patients with organ involvement, where their use should be more aggressive (25). A review of the patient’s diagnostic and therapeutic course reveals that during the early phase, when only skin purpura was present, high-dose corticosteroid pulse therapy was not administered. The patient also did not seek medical attention immediately after the onset of abdominal pain. It was only after the diagnosis of concurrent pancreatitis and esophageal mucosal exfoliation that high-dose corticosteroid pulse therapy combined with immunoglobulin treatment was initiated. Unfortunately, the patient experienced massive gastrointestinal (GI) bleeding and responded poorly to medical management. Ultimately, the patient succumbed to disseminated intravascular coagulation (DIC) secondary to severe bleeding. The patient’s condition progressed rapidly. Although glucocorticoids and immunoglobulins were administered, they failed to effectively reverse the uncontrolled immune response. Novel agents—such as the oral targeted-release formulation of budesonide, B cell–directed agents, and complement pathway inhibitors—have demonstrated favorable outcomes in some clinical trials and may offer additional therapeutic options for the management of such patients in the future (26). This case underscores the potential for GI involvement in IgAV to present in a highly insidious manner, highlighting the need for careful diagnostic reflection. Although abdominal CT is the routine first-line examination for acute abdomen, clinicians should remain vigilant for possible GI lesions in patients with IgAV presenting with abdominal pain. This rare, catastrophic case reminds us that early and thorough gastrointestinal endoscopy is essential to identify potential life-threatening complications in the digestive tract.

## 4 Conclusion

This case highlights a rare and catastrophic complication of immunoglobulin A vasculitis (IgAV) presenting with acute esophageal necrosis and pancreatitis. Clinicians should maintain a high index of suspicion for unusual gastrointestinal (GI) involvement in patients with IgAV presenting with acute abdomen, and early imaging and endoscopic evaluation may be warranted. Prompt initiation of immunosuppressive therapy could potentially alter outcomes; however, further case accumulation is needed to better guide management.

## Data Availability

The original contributions presented in the study are included in the article/**Supplementary Material**. Further inquiries can be directed to the corresponding author.
