# Supplementary material for: Case Report: Severe gastrointestinal complications in adult IgA vasculitis: a fatal case of acute esophageal necrosis
Source: Front Immunol. 2025 Oct 21;16:1601700. doi: 10.3389/fimmu.2025.1601700 (PMC12584157; doi:10.3389/fimmu.2025.1601700)
Supplement: Supplementary file 1 [file Table1.docx]

**
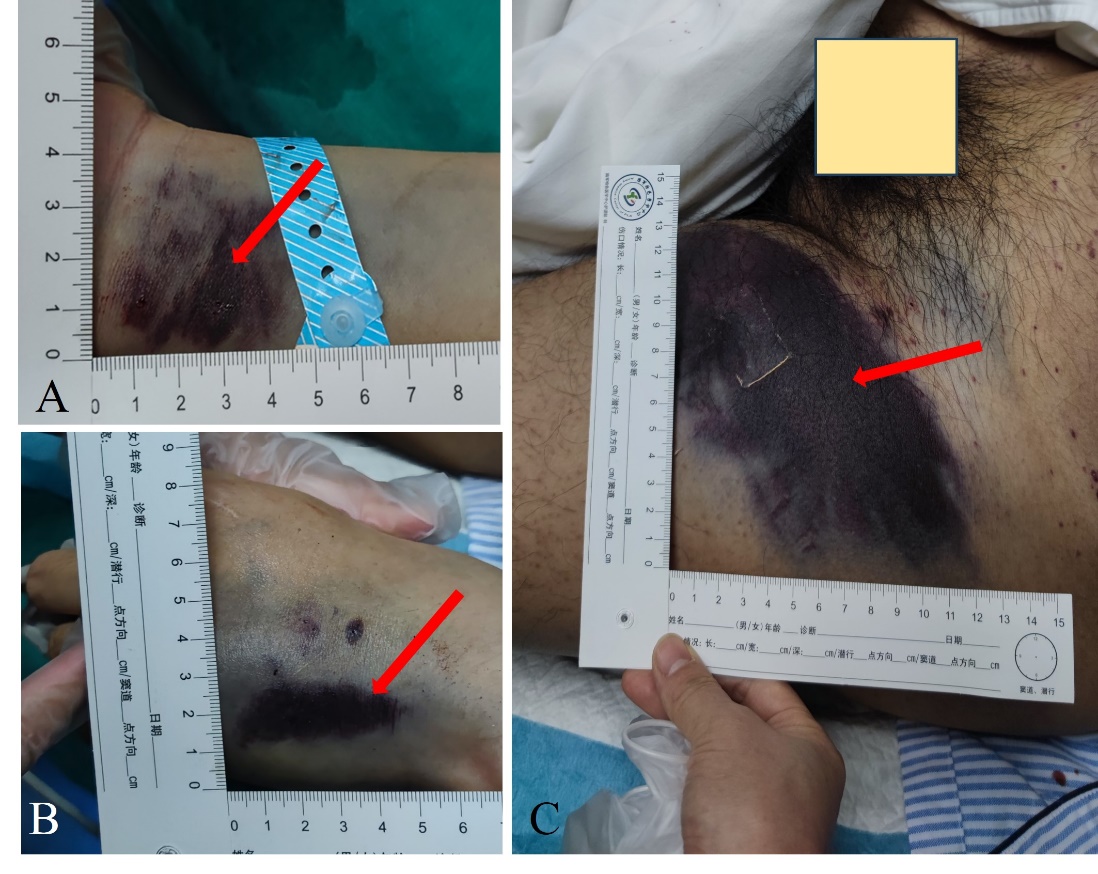
**

**Supplementary Figure 1.** Large patches of purpura on the wrist, back of the hand, and right inguinal area.
